# Preclinical Assessment of the Treatment of Second-Stage African Trypanosomiasis with Cordycepin and Deoxycoformycin

**DOI:** 10.1371/journal.pntd.0000495

**Published:** 2009-08-04

**Authors:** Suman K. Vodnala, Marcela Ferella, Hilda Lundén-Miguel, Evans Betha, Nick van Reet, Daniel Ndem Amin, Bo Öberg, Björn Andersson, Krister Kristensson, Hans Wigzell, Martin E. Rottenberg

**Affiliations:** 1 Department of Microbiology, Tumor and Cell Biology, Karolinska Institute, Stockholm, Sweden; 2 Department of Cell and Molecular Biology, Karolinska Institute, Stockholm, Sweden; 3 Tropical Diseases Research Center, Ndola Central, Ndola, Zambia; 4 Institute of Tropical Medicine, Antwerp, Belgium; 5 Department of Neurosciences, Karolinska Institute, Stockholm, Sweden; 6 Medivir AB, Huddinge, Sweden; Foundation for Innovative New Diagnostics (FIND), Switzerland

## Abstract

**Background:**

There is an urgent need to substitute the highly toxic compounds still in use for treatment of the encephalitic stage of human African trypanosomiasis (HAT). We here assessed the treatment with the doublet cordycepin and the deaminase inhibitor deoxycoformycin for this stage of infection with *Trypanosoma brucei (T.b.)*.

**Methodology/Principal Findings:**

Cordycepin was selected as the most efficient drug from a direct parasite viability screening of a compound library of nucleoside analogues. The minimal number of doses and concentrations of the drugs effective for treatment of *T.b. brucei* infections in mice were determined. Oral, intraperitoneal or subcutaneous administrations of the compounds were successful for treatment. The doublet was effective for treatment of late stage experimental infections with human pathogenic *T.b. rhodesiense* and *T.b. gambiense* isolates. Late stage infection treatment diminished the levels of inflammatory cytokines in brains of infected mice. Incubation with cordycepin resulted in programmed cell death followed by secondary necrosis of the parasites. *T.b. brucei* strains developed resistance to cordycepin after culture with increasing concentrations of the compound. However, cordycepin-resistant parasites showed diminished virulence and were not cross-resistant to other drugs used for treatment of HAT, i.e. pentamidine, suramin and melarsoprol. Although resistant parasites were mutated in the gene coding for P2 nucleoside adenosine transporter, P2 knockout trypanosomes showed no altered resistance to cordycepin, indicating that absence of the P2 transporter is not sufficient to render the trypanosomes resistant to the drug.

**Conclusions/Significance:**

Altogether, our data strongly support testing of treatment with a combination of cordycepin and deoxycoformycin as an alternative for treatment of second-stage and/or melarsoprol-resistant HAT.

## Author Summary

There is an urgent need to substitute the highly toxic arsenic compounds still in use for treatment of the encephalitic stage of African trypanosomiasis, a disease caused by infection with *Trypanosoma brucei*. We exploited the inability of trypanosomes to engage in *de novo* purine synthesis as a therapeutic target. Cordycepin was selected from a trypanocidal screen of a 2200-compound library. When administered together with the adenosine deaminase inhibitor deoxycoformycin, cordycepin cured mice inoculated with the human pathogenic subspecies *T. brucei rhodesiense* or *T. brucei gambiense* even after parasites had penetrated into the brain. Successful treatment was achieved by intraperitoneal, oral or subcutaneous administration of the compounds. Treatment with the doublet also diminished infection-induced cerebral inflammation. Cordycepin induced programmed cell death of the parasites. Although parasites grown *in vitro* with low doses of cordycepin gradually developed resistance, the resistant parasites lost virulence and showed no cross-resistance to trypanocidal drugs in clinical use. Our data strongly support testing cordycepin and deoxycoformycin as an alternative for treatment of second-stage and/or melarsoprol-resistant HAT.

## Introduction

Human African trypanosomiasis (HAT), also known as sleeping sickness, is a neglected tropical infectious disease that has re-emerged in sub-Saharan Africa in the late 1900^ties^ [1],[2]. The disease is caused by subspecies of the protozoan parasite *Trypanosoma brucei* and is transmitted by the blood-sucking tsetse fly. Following a hemo-lymphatic stage with waves of parasitemia, the nervous system is involved manifested as a leukencephalitis, invariably lethal if left untreated. Drugs, which are effective at an early stage of the disease, but poorly penetrate the blood-brain barrier, are ineffective for the second stage. For treatment of the second encephalitic stage of HAT, arsenic compounds such as melarsoprol, which are associated with severe and even lethal side-effects, are still widely used. Moreover, there has been an alarming increase in melarsoprol-refractory sleeping sickness cases [3]. Dl-α-difluoromethylornithine (DFMO) is used for treating the West African form of HAT, caused by *T.b. gambiense*. However, this drug is given by intravenous injections, is expensive, and is not effective against *T.b. rhodesiense* that causes the East African form of HAT. Since the development of new drugs for HAT is not likely to occur in the immediate future, the strategy of testing drugs approved or in clinical use for other diseases should be pursued in order to identify less toxic or alternative drugs to cure second stage HAT [4].

In contrast to most mammalian cells, trypanosomes cannot synthesize purines *de no*vo. Instead they depend on the salvage pathway of nucleosides from the body fluids of the host [5]. The inability of trypanosomes to engage in *de novo* purine synthesis has been exploited as a therapeutic target. The trypanocidal potential of cordycepin (3′-deoxyadenosine), a metabolite from the fungi *Cordyceps spp.*, was noted in experiments performed in the 1970's [6],[7]. However, administration of cordycepin did not result in a complete cure from infection [8], since cordycepin is rapidly converted to inactive 3′-deoxyinosine by adenosine deaminase (ADA) *in vivo* [9]. Deoxycoformycin is an ADA inhibitor that can prevent degradation of cordycepin, and have come into use in combination with cordycepin for the treatment of certain malignant tumors in humans, e.g. leukemia and melanoma [10].

Cordycepin is probably taken up by trypanosomes through transporters. Nucleoside transport systems, P1 and P2, are able to concentrate cordycepin inside the cells and has turned out to play an important role in the uptake of trypanocides [11]. Adenosine is salvaged through a two-step process in *T. brucei* where its intracellular cleavage to adenine is followed by phosphoribosylation to AMP, or by a high affinity adenosine kinase [12],[13]. Cordycepin is also a potent inhibitor of the mammalian poly-A polymerase, and has been shown to hamper the activity of nucleoside-stimulated protein kinase activity in *Trypanosoma sp.* [14].

We have previously shown that intraperitoneal (i.p.) injection of cordycepin, together with coformycin or deoxycoformycin, can cure *T.b. brucei* infections in mice [15]. Treatment was effective, as recorded by the absence of parasitemia up to three weeks after the end of the treatment, even when instituted after the trypanosomes had penetrated into the brain parenchyma. The aim of the present work was to further improve treatment strategies with nucleosides, and gain knowledge on the effect of these compounds on the parasite. We could show that cordycepin is the best candidate drug selected after screening a library of 2200 nucleoside analogues for parasite differential toxicity. The minimal inhibitory concentrations of cordycepin and deoxycoformycin and the minimal number of doses required for effective treatment were then determined in mice infected with *T.b. brucei.* Cordycepin and deoxycoformycin treatment did also cure second stage infection with human pathogenic *T.b. gambiense* and *T.b. rhodesiense,* and oral administration of the duplet when combined with a proton pump inhibitor was effective. Cultivation of *T.b. brucei* with low doses of cordycepin allowed resistance to cordycepin to develop, but resistant parasites lost virulence, and no cross-resistance between cordycepin and melasoprol. suramin and pentamidine was noted. Importantly treated mice showed diminished levels of pro-inflammatory cytokines in the brain.

Altogether, our findings encourage the possible use of cordycepin and deoxycoformycin as an alternative for treatment of second stage HAT or melasoprol-resistant HAT.

## Methods

### Reagents

Cordycepin, suramin, pentamidine, omeprazole and EHNA (erythro-9-(2-hydroxy-3-nonyl)adenine) were all purchased from Sigma (St Louis, MO). Deoxycoformycin (pentostatin) was a kind gift of R. McCaffrey (Brigham & Women's Hospital, Harvard Medical School, Boston, Massachusetts). Melarsoprol (Arsobal) was a gift from P. Simarro (WHO/NTD, Geneve, Switzerland). Unless otherwise indicated, all reagents were diluted in PBS, fractioned and stored at −20°C until further use.

The library of nucleoside analogs used consisted of compounds design to be inhibitors of various viral and microbial enzymes and is property of Medivir AB.

### Mice

BALB/c, C57Bl/6 and RAG1^−/−^ [16] (backcrossed on a C57Bl/6 background) male mice, 8–10 weeks old at the beginning of the experiments, were used. Mice were kept with food and water *ad libitum* under specific pathogen-free conditions. All experiments were conducted following protocols that received institutional approval and authorization by the Stockholm Region's animal protection committee.

### Parasites

*T.b. brucei* (AnTat1.1E), *T.b. rhodesiense* (STIB 851) and *T.b. gambiense* (MBA) were kindly provided by P. Büscher (Institute of Tropical Medicine, Antwerp, Belgium). Tbat1 null parasites constructed by sequential homologous recombination of *T.b. brucei* Lister 427 [17], were kindly provided by P. Maser (University of Bern, Switzerland). Lister 427 parasites were used as controls. Parasitemia was measured every 2 or 3 day in tail vein blood. Body weight and morbidity were regularly recorded.

### Parasite culture and viability

Bloodstream forms of *T.b. brucei, T.b. gambiense* and *T.b. rhodesiense* freshly isolated from infected C57BL/6 mice (3 days post infection with 1×10^7^ parasites/mouse) were separated by DEAE-cellulose chromatography under sterile conditions [18]. *T. brucei* parasites were incubated in D-MEM containing 10% heat-inactivated calf serum, 28 mM HEPES, 0.14% glucose, 1.5% NaHCO_3_, 2 mM L-glutamate, 0.14 mg/ml gentamycin, 0.3 mM dithiothreitol, 1.4 mM sodium pyruvate, 0.7 mM L-cysteine, 28 µM adenosine, 14 µM guanosine at 37°C.

To measure drug sensitivity, 2.5×10^4^ parasites were cultured in 96 flat-bottomed well culture plates with serial drug dilutions for 72 h at 37°C. Cultures (100 µl) were incubated for 2 h with 10 µl of WST-1 reagent (Roche, Mannheim, Germany). Viability was measured by the conversion of WST-1 reagent to formazan, recorded by multiwell scanning spectrophotometer at an excitation wavelength of 450 nm. A direct correlation between WST-1 reagent oxidation and parasite numbers was confirmed (data not shown).

### Immunohistochemistry

Mice were deeply anesthetized with isoflurane, sacrificed and brains were dissected and snap frozen. To examine presence of trypanosomes within and outside the blood vessels in the brain, cryostat sections were cut and immunolabelled with anti-AnTat1.1 VSG (1∶5.000; obtained from N. van Meirvenne, Institute of Tropical Medicine, Antwerp, Belgium) and goat polyclonal anti-glucose transporter 1 (1∶40; GLUT-1, Santa Cruz Biotechnology, Santa Cruz, CA, USA) as described previously [19].

### Biophotonic measurement of parasite dissemination

Stable recombinant *Renilla* luciferase expressing parasites were generated as recently described (F Claes, submitted). The use of such luciferase tagged *T. brucei* for real time studies of parasite dynamics was validated **in vitro** and **ex vivo** (F. Claes, *PLoS NTD* in press). BABLB/c mice were infected i.p. with 2×10^3^ luciferase tagged *T.b. brucei* AnTat1.1. At different days after infection, mice were anesthetized with 2.3% isoflurane, injected intraperitoneally with 100 µL of coelenterazine (2 µg/µl dissolved in methanol) (Synchem) diluted with 90 µL PBS pH 7, and light emission in photons/second/cm^2^/steradian (p/sec/cm^2^/sr) was recorded in an IVIS Imaging System 100 (Xenogen LifeSciences) and Living Image 2.20.1 software (Xenogen) for 180 seconds. Measurements started 3–5 minutes after substrate injection to allow the spread of the coelenterazine.

### Markers for apoptosis

#### DNA content

Briefly, following a 30 min permeabilization of trypanosomes in 10 mM phosphate buffer containing 6 µM digitonin, nuclei were stained with a 10 µg/ml propidium iodide solution in 10 mM phosphate buffer and kept on ice until measurement by flow cytometry.

#### DNA fragmentation

DNA fragmentation was assessed by using the terminal deoxynucleotidyltransferase-mediated dUTP nick end labeling method (Roche) as described [20]. Analysis was performed on a Becton Dickinson FACScan, and data were acquired on a minimum of 10,000 cells per sample.

#### Phosphatidylserine exposure

Exposed phosphatidylserine was detected on the outer membrane of cells using Annexin-V-Fluor (Roche) following instructions by the manufacturer. Fluorescence was measured using FACS analysis as described before [21].

#### Necrosis

In order to analyze disruption of the plasma membrane after cordycepin treatment, nuclei were stained with propidium iodide (5 µg/ml) in the absence of a cell permeant and analyzed by flow cytometry.

### Real time RT-PCR

Gene transcripts of several pro-inflammatory cytokines were quantified in brains from cordycepin-treated and PBS-treated, uninfected and infected mice by real time PCR. Total RNA was extracted from half of the fresh frozen brains, reverse-transcribed, and the transcripts levels quantified on an ABI Prism 7000 sequence detection system (Applied Biosystems) as described previously [16]. Ten-fold dilutions of a cDNA sample were amplified to control amplification efficiency for each primer pair. Thereafter, the threshold cycle value, Ct (the fractional cycle number at which the fluorescence reaches a fixed threshold) was obtained for all cDNA samples. The amount of transcripts of individual animal samples (n = 4 per group) was normalized to HPRT (ΔC_t_). The relative amount of target gene transcripts was calculated using the 2^−ΔΔCt^ method as described [22]. These values were then used to calculate the mean and standard error of the relative expression of the target gene mRNA in the brain of uninfected and infected mice.

### Parasite DNA amplification

Total DNA was extracted from brains from treated and untreated mice using a Dneasy tissue kit (Qiagen, Duesseldorf, Germany). The amount of parasite DNA was quantified by real time PCR as described above using *senso latu T. brucei* primers [23]. The concentration of DNA in each sample was measured in a spectrophotometer. A correlation between parasite numbers and quantification of parasite DNA by real time PCR was determined.

### Candidate genes cloning and sequencing

Total DNA from parental and resistant strains was extracted using the DNeasy tissue kit (Qiagen). Candidate genes suspected to be involved in the resistance to cordycepin were PCR amplified from total DNA using forward and reverse specific primers designed over the following annotated genes by the *T. brucei* genome project (Table S1): Adenosine kinase, Adenine phosphoribosyltransferase (APRT), Hypoxanthine-guanine phosphoribosyltransferase (HGPRT), Poly(A) polymerase, Hexose Transporter 1 (HT1), Aminopurine Transporter (AT1 or P2), P1 family of nucleoside transporters (NT2, NT4, NT6 and NT12). PCR products were A-tailed and ligated into pGEM T-vector (Promega, Madison, WI). A set of 6 to 12 positive colonies for each gene was sequenced using the DYEnamic ET dye terminator kit and a Megabase sequencer (GE) with m13 forward and reverse primers, and when needed gene specific internal primers. Sequences were assembled and analyzed using phred-phrad and consed. Sequence analysis and comparisons between parental and resistant strains and genome-annotated versions were performed with ClustalW (www.ebi.ac.uk) and Blast2seq (www.ncbi.nlm.nih.gov). Single nucleotide polymorphisms between clones were taken into account.

## Results

### Screening of a nucleoside compound library

We have previously suggested that cordycepin could be used for treatment of second stage African trypanosomiasis. Whether other nucleoside analogues could have a more selective toxic effect on *T.b. brucei* was first studied. For this purpose we screened a library of 2200 drug-like nucleosides compounds for toxicity against *T.b. brucei* AnTat1.1. The screening revealed 14 hits showing toxicity for *T.b. brucei* at less than 1 µM concentration. Ten of these compounds were found to also be toxic for mammalian cells at these concentrations. One of the remaining compounds was identified as cordycepin. A second one was tubercidin, a nucleoside previously shown to inhibit the respiratory chain in *T.b. brucei*, but that induced adaptation of parasites to a glucose-independent metabolism [24]. The two other compounds were diamines, N′trityl-1,2-diaminoethane hydrobromide and N-trityl-1,3-siaminopropane acetate, included in the library as intermediates used in the chemical synthesis of nucleoside analogues.

**Figure 1:**
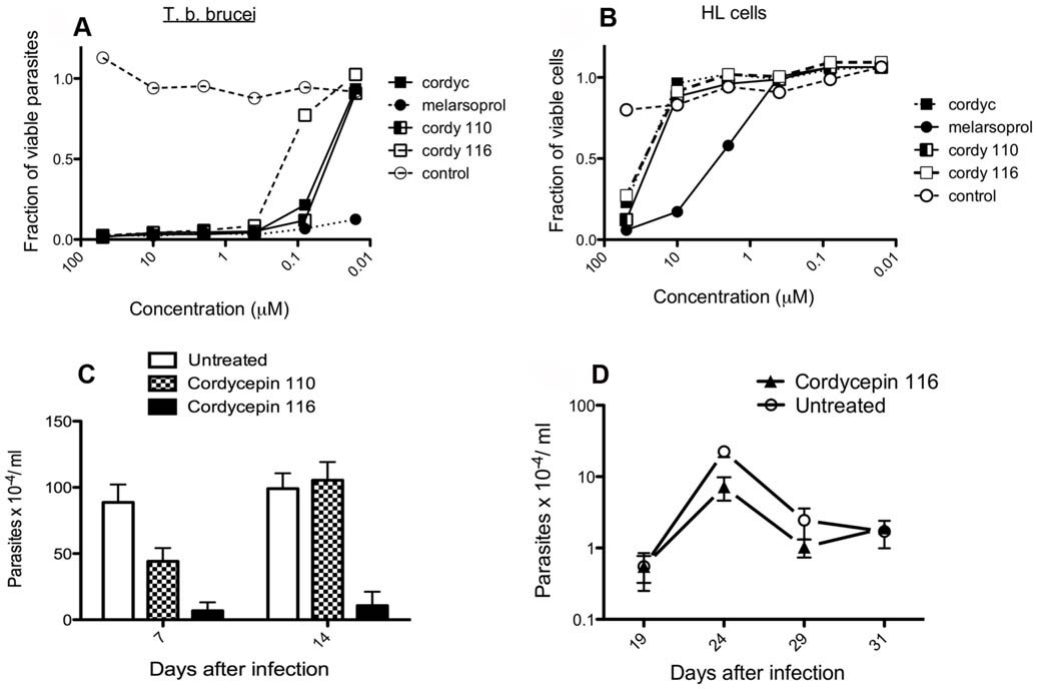
Trypanocidal activity of cordycepin and cordycepin derivatives. A, B *T.b. brucei* or HL cells were incubated in vitro with different concentrations of the indicated compounds for 48 h. The relative OD values with respect to untreated cells after WST-1 A) or crystal violet staining B) is shown. The IC_50_ of cordycepin and derivatives against *T. b. brucei* AnTat1.1 were cordycepin: 32 nM; cordycepin 110: 26 nM and cordycepin 116: 128 nM. The IC_50_ for HL cells of the same compounds was in the range of 50–100 µM. C, D Mice were infected i.p. with 2×10^3^ *T.b. brucei.* Parasitemia levels in mice treated with 2 mg/kg/d of the indicated cordycepin derivatives for 2 days starting at the day of infection C) or 20 days after infection D) were measured. A control infected group was left untreated. The mean parasitemia and the standard error of the mean are depicted.

Subsequently, the trypanocidal activity of cordycepin was compared with that of adenosine deaminase resistant nucleoside analogues. Such nucleosides could potentially act as stand-alone drugs in treatment of *T.b. brucei* infections. Three out of 22 tested compounds showed at least 3 log higher toxicity for *T.b. brucei* as compared to mammalian HL-cells (Figure 1A–B and data not shown). One is 2-fluorodeoxyadenosine and the other are two synthetic cordycepin derivatives: 6-N-(phenylthio)-methyl-cordycepin (cordycepin 110) and 6-N-(4-N-acethylbenzylthio)-methyl-cordycepin (cordycepin 116). As expected, deoxycoformycin was not toxic for *T.b. brucei* (data not shown).

The two synthetic derivatives of cordycepin were tested *in vivo*. Cordycepin 116, but not cordycepin 110, showed anti-trypanosomal effect during the early stage of infection (Figure 1C). However, no curative effect was observed when 2 mg/kg/d cordycepin 116 was inoculated during 10 days in the absence of deoxycoformycin and starting 20 days after infection with *T.b. brucei* (Figure 1D). Thus, we decided to restrict our further evaluation of the effects of treatment to cordycepin during infection with *T. brucei* on the host and the parasite.

### Determination of minimal effective cordycepin concentrations

Experiments were then performed to confirm, using more extended protocols, the curative effect of cordycepin and deoxycoformycin on the second stage murine infection with *T.b. brucei*. In order to detect the presence of remnant parasites in the tissues of treated animals, immunodeficient RAG-1^−/−^ mice were inoculated i.p. with blood or brain tissue homogenates of infected and treated mice. A double immuno-labelling method using antibodies against trypanosomes and cerebral endothelial cells was used to detect parasites in the brain parenchyma. Stained sections from brain of treated animals (4 per animal) were analyzed. Parasite DNA in whole blood or brain tissues was measured by real time PCR.

In a first experiment we showed that none of the C57Bl/6 mice (n = 9) treated i.p. with 2 mg/kg body weigth (kg)/day (d) cordycepin and 0.2 mg/kg/d deoxycoformycin for 7 consecutive days, starting at day 20 after infection with 2×10^3^ *T.b. brucei.* displayed parasites at 85 days of infection (65 days after treatment). No parasites were detected in the blood of cordycepin- and deoxycoformycin-treated mice. On the contrary, although no parasites were detected directly after treatment with 20 mg/kg suramin, the presence of parasites was recorded at least once in the blood of all suramin-treated mice before sacrifice (65 days after treatment), although only in a fraction of mice at a given time.

Whereas all RAG1^−/−^ mice inoculated with blood or brain homogenates from suramin-treated mice showed parasites early after infection, no parasites were detected in mice inoculated with either blood or brain homogenates from cordycepin- and deoxycoformycin-treated mice. Parasite DNA could not be amplified and parasite antigens were not detected in the brain tissues of cordycepin- and deoxycoformycin-treated mice. Infected and non-treated mice showed signs of morbidity before day 32 after infection. No mortality was recorded in the cordycepin-treated group and 1 out of 8 suramin-treated mice died 79 days after infection.

**Figure 2:**
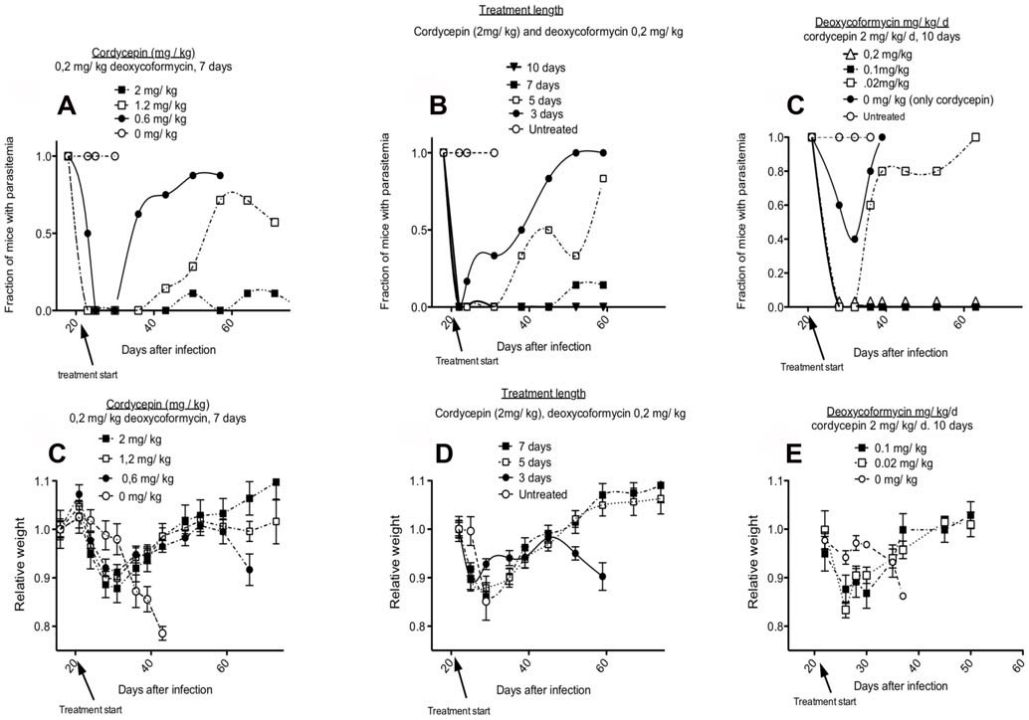
Minimal effective doses of cordycepin and deoxycoformycin for treatment of late stage infection with *T.b. brucei*. A, D Different concentrations of cordycepin with a 0.2 mg/kg/d deoxycoformycin were inoculated i.p. for 7 days into mice starting 20 days after infection with 2×10^3^ *T.b. brucei*. B, E Different number of doses of 2 mg/kg cordycepin and 0.2 mg/kg/d deoxycoformycin were inoculated starting 20 days after infection with 2×10^3^ *T. brucei*. C, F Different doses of deoxycoformycin were inoculated i.p. daily for 10 days with 2 mg/kg cordycepin starting 20 days after infection with *T. brucei*. A–C The fraction of mice with detectable parasitemia is depicted. D–F The mean of weight change in relation to that before infection in each group±standard error the mean is shown.

In a second experiment the minimal effective concentration of cordycepin was determined. For this purpose, we compared the treatment of mice given different doses of cordycepin and a constant dose of deoxycoformycin. Mice were inoculated with 2, 1.2 and 0.6 mg/kg/d cordycepin plus 0.2 mg/kg/d deoxycoformycin for 7 days starting at day 20 after infection. Whereas the trypanocidal effect was evident in all groups of mice (n = 9), a dose-dependent effect was observed, with doses lower than 2 mg/kg/d not sufficient to completely clear parasitemia (Figure 2A). One out of 9 mice inoculated with 2 mg/kg/d cordycepin showed parasites before 80 days after infection.

Next, groups of mice (n = 7) were dosed with 2 mg/kg/d cordycepin and 0.2 mg/kg/d deoxycoformycin during 3, 5 or 7 days starting 20 days after infection. Again, a dose-dependent trypanocidal effect was observed also in this experiment. Five or three doses were not enough to completely clear infection, whereas in the group treated with 7 doses only one animal remained infected (Figure 2B). Since, a total of 2 of out of 25 mice in the different experiments treated with 2 mg/kg/d cordycepin and 0.2 mg/kg/d deoxycoformycin for 7 days remained infected after treatment, we concluded that this treatment is on the brink of the minimal effective dose for clearance. We then increased the number of doses used for treatment. When mice (8 per group) were treated for 10 or 15 days with 2 mg/kg/d cordycepin and 0.2 mg/kg/d deoxycoformycin starting at day 20 after infection, a complete curative effect was achieved in two independent experiments (33 mice together) (Figure 2B and data not shown).

The minimal effective concentration of deoxycoformycin required for treatment was subsequently evaluated. For this purpose, mice (8 per group) were treated for 10 days with 2 mg/kg/d cordycepin and different concentrations of deoxycofomycin starting 20 days after infection with 2×10^3^ *T.b. brucei.* Treatment with either 0.1 or 0.2 mg/kg/d deoxycoformycin resulted in complete cure of second stage infection, while no curative effect was observed when 0.02 mg/kg/d deoxycoformycin was used for treatment (Figure 2C). We also tested whether EHNA, another ADA inhibitor, could replace deoxycoformycin in treatment of late stage infections. Similar transient decreases in parasites levels in the blood were observed when 2 mg/kg/d cordycepin was inoculated in presence or absence of 0.2 mg/kg/d EHNA (data not shown), suggesting that EHNA did not protect cordycepin from degradation.

Starting treatment with cordycepin and deoxycoformycin at 20 days after infection resulted in loss of weight of infected mice. Surprisingly, toxicity was largely independent of the dose of cordycepin and deoxycoformycin used, since mice treated either for 15, 10, 7, 5 or 3 days with 2 mg/kg/d cordycepin and 0.2 mg/kg/d deoxycoformycin or treated with 7 doses of 2, 1.2 or 0.6 mg/kg/d cordycepin and 0.2 mg/kg/d deoxycoformycin all showed similar weight loss (Figure 2D, E and data not shown). All mice recovered weight after the treatment. No other sign of morbidity was noted. Importantly, weight loss was not observed in uninfected mice treated with 2 mg/kg/d cordycepin and 0.2 mg/kg/d deoxycoformycin (data not shown). Thus, the treatment toxicity required simultaneous presence of an established infection.

**Figure 3:**
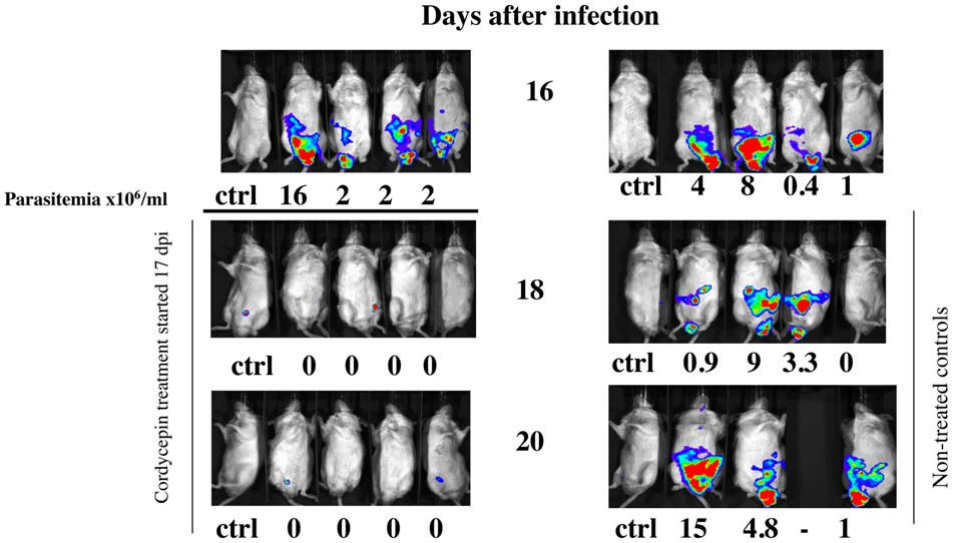
Dynamics of infection with *T.b. brucei* AnTat 1.1 infection in mice treated or not with cordycepin and deoxycoformycin, visualized by bioluminescence imaging. Parasite load in BALB/c male mice injected i.p. with 2×10^4^ luciferase recombinant *T.b. brucei* AnTat 1.1 was assessed daily by biophotonic emission determination as indicated in material and methods. Parasitemia levels were registered in parallel.

Real-time imaging of biophotonic emission provides a fast method to evaluate parasite dissemination *in vivo*. BALB/c mice were infected with 2×10^3^ luciferase-tagged *T.b. brucei* AnTat1.1. Bioluminescent images showed a systemic spread of infection. No light emission was observed from mice treated with the doublet during late stage of infection (Figure 3), confirming the efficiency of treatment with cordycepin and deoxycoformycin.

### Oral and subcutaneous administration of cordycepin and deoxycoformycin

**Figure 4:**
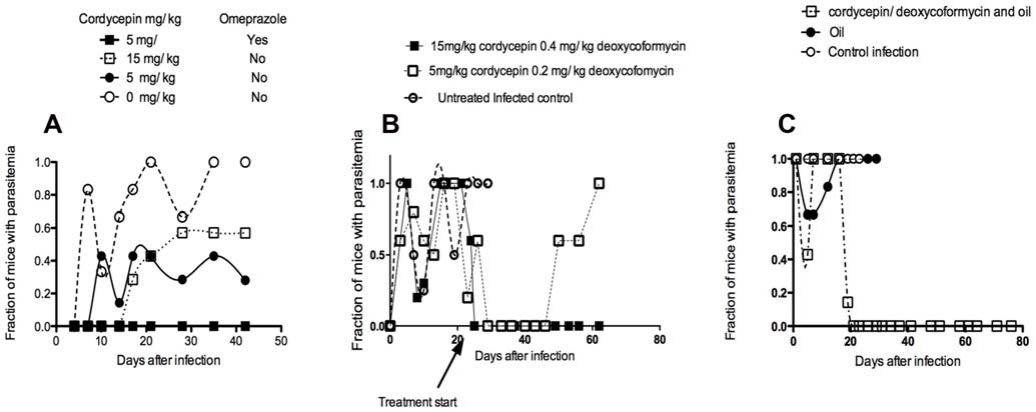
Curative effect of oral and subcutaneous inoculation of cordycepin and deoxycoformycin on early and late stage infection with *T.b. brucei*. A Mice were orally administered with the indicated doses of cordycepin with 0.2 mg/kg/d deoxycoformycin in presence or absence of omeprazole, daily 3 times starting at the day of infection with 2×10^3^ *T.b. brucei*. B Mice were orally administered with the indicated doses of cordycepin with deoxycoformycin in presence of omeprazole, daily 10 times starting at the day 20 after infection with 2×10^3^ *T.b. brucei*. C Mice were inoculated s.c. daily for 10 days with 2 mg/kg cordycepin and 0.2 mg/kg deoxycoformycin in vegetable oil or with oil alone. The fraction of mice with detectable parasitemia is indicated in A, B and C.

The customary i.v. administration of trypanocidal drugs requires trained medical personnel, and specialized equipment and materials. Oral administration of these compounds would facilitate their use in patients. The effect of oral administration of cordycepin and deoxycoformycin in the outcome of murine infection with *T. b. brucei* was thus tested. We found that oral administration of 5 or 15 mg/kg/d cordycepin with 0.2 mg/kg/d deoxycoformycin cured approximately 50% of the treated mice when treatment was started at the day of infection and continued for 3 days. While oral administration of cordycepin has been shown to be an effective anti-tumoral agent [25], deoxycoformycin has a well described acid-lability that has hampered its use in oral formulations. Cordycepin and deoxycoformycin were then administered with omeprazole, a proton pump inhibitor, to reduce acid concentration in the stomach and thereby allow absorption of enhanced concentrations of the ADA inhibitor. Daily treatment of mice at the day of infection with cordycepin plus deoxycoformycin in presence of omeprazole for 5 days resulted in cure of the infection of all (n = 6) treated mice (Figure 4A). We then tested if oral administration of the doublet was effective for treatment of second stage infection with *T. b. brucei.* We found that oral administration of 15 mg/kg/d cordycepin and 0.4 mg/kg/d deoxycoformycin, but not 5 mg/kg/d cordycepin and 0.2 mg/kg/d deoxycoformycin, for 10 days starting 20 days after infection had a complete curative effect (Figure 4 B).

Subcutaneous treatment with 2 mg/kg/d and 0.2 mg/kg/d deoxycoformycin diluted in 100 µl of vegetable oil daily for 10 days starting on day 20 after infection with *T.b. brucei* was also effective in curing late stage infection of treated animals. Administration of 4 mg/kg/d cordycepin and 0.2 mg/kg/d deoxycoformycin s.c. every other day failed to cure all treated mice, suggesting that strategies improving pharmacokinetics of the doublet might improve treatment efficiency (Figure 4C).

### Cordycepin and deoxycoformycin treatment of infections with human pathogenic *T. brucei* isolates

**Figure 5:**
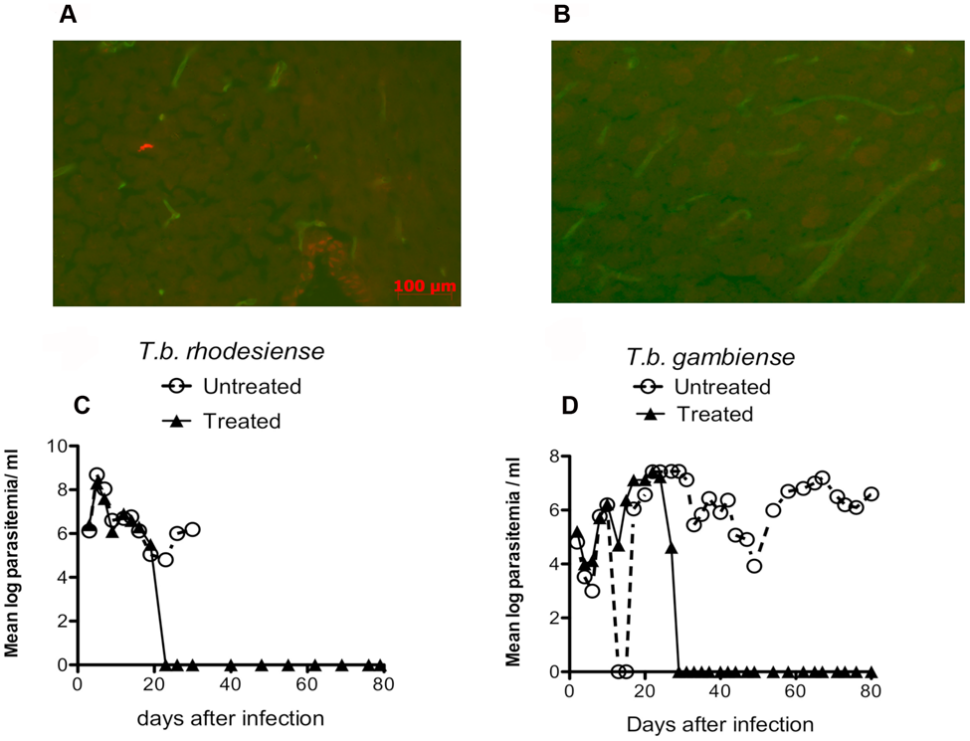
Curative treatment with cordycepin and deoxycoformycin on late stage experimental *T.b. rhodesiense* infection. Mice were inoculated i.p. with 2×10^4^ *T.b. rhodesiense*. Increasing density of parasite immunostaining in the brain parenchyma was observed at 15, 21 and 28 days after infection (data not shown). A representative section of parasite immunostaining in the brain of mice at day 21 after infection is shown in A. Note in green labeling of brain endothelial cells and in red, *T. brucei*. An uninfected mouse brain section incubated with both antibodies is shown as a negative control in panel B. C Groups of mice were infected with *T.b. rhodesiense* and inoculated i.p. daily for 10 days with 2 mg/kg/d cordycepin and 0.2 mg/kg/d deoxycoformycin starting 21 days after infection, or left untreated. The mean log parasitemia of cordycepin and deoxycoformycin treated *T.b. rhodesiense* infected mice is indicated. D The mean log parasitemia from groups (n = 7 animals per group) infected with 2×10^5^ *T.b. gambiense* and treated with cordycepin and deoxycoformycin for 10 days starting 30 days after infection, or left untreated is depicted.

We then studied whether late stage infection with human pathogenic strains of *T. brucei* can be treated with cordycepin and deoxycofomycin. Two weeks after infection with 10^4^ *T.b. rhodesiense* STIB 851 isolate, parasites were detected in the brain parenchyma. Parasite density in the brain parenchyma increased at later times of infection (Figure 5A, B). Parasitemia levels were low or undetectable compared to *T.b. brucei* AnTat1.1 (Figure 5 C), but mice lost weight and showed signs of morbidity at 35–40 days after infection (data not shown). Ten days of dosing of 2 mg/kg/d cordycepin and 0.2 mg/kg/d deoxycoformycin treatment cured infection in mice as evaluated by real time PCR and inoculation of lysates of brains from mice 65 days after treatment to naïve immunodeficient mice (data not shown).

The effect of cordycepin and deoxycoformycin administration on the outcome of infection with *T.b. gambiense* was then evaluated. *T.b.gambiense* isolates were also susceptible to cordycepin *in vitro* (data not shown). Whereas mice inoculated i.p. with 2×10^5^ *T.b. gambiense* (MBA stabilates) showed parasitemia up to at least 70 days after infection, mice inoculated 2×10^3^ or 2×10^4^ *T.b. gambiense* showed no parasites in the blood. In contrast to *T.b. rhodesiense*, morbidity was only observed in *T.b. gambiense*-infected mice at the time of sacrifice (80 days after infection) with small numbers of parasites observed in brain parenchyma, with elevated parasite densities observed only in the septum (data not shown). Treatment of *T.b. gambiense* infected mice with 2 mg/kg/d cordycepin and 0.2 mg/kg/d deoxycoformycin for 10 days, starting at 30 days after infection, when parasites where first detected in the brain parenchyma, completely eliminated parasitemia for at least 50 days after treatment (Figure 5 D).

Thus, treatment with cordycepin and deoxycoformycin was effective in treatment of late stage infection with both human pathogenic subspecies.

### Cordycepin and deoxycoformycin treatment diminished accumulation of pro-inflammatory cytokines in the brain

**Figure 6:**
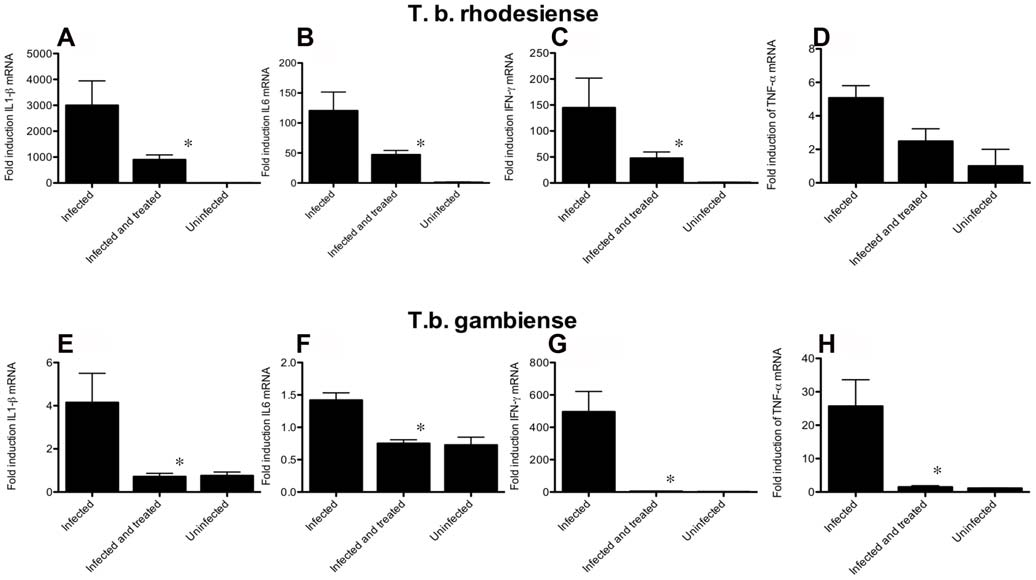
Levels of pro-inflammatory cytokine transcripts in brains of T.b. rhodesiense and *T.b. gambiense*-infected, cordycepin and deoxycoformycin-treated mice. Total RNA was extracted from brain tissues of mice 30 days after inoculation with *T.b. rhodesiense* A–D) or 80 days after inoculation with *T.b. gambiense* E–H), treated or not with 2 mg/kg/d cordycepin and 0.2 mg/kg/d deoxycoformycin starting 20 or 30 days after infection respectively, converted into cDNA and pro-inflammatory mRNA accumulation was measured by real time PCR. The number of moles of cytokine per mole of HPRT in samples from individual mice (4–6 per group) was calculated and the fold increase of IL-1 A, E), IL-6 B, F), IFN−γ C, G), TNF-α D, H) in cordycepin and deoxycoformycin treated or untreated infected mice with respect to levels in brains of one of the uninfected mice depicted. *Differences with untreated, infected group are significant (p<0.05 Student t test).

Brains from *T.b. rhodesiense*-infected mice treated with cordycepin and deoxycoformycin starting 20 days after infection contained diminished levels of IFN-γ, IL-1β, IL-6 and TNF-α mRNA compared to untreated infected controls when measured 10 days after treatment (Figure 6A–D). Likewise, brains from mice infected with *T.b. rhodesiense* and treated with cordycepin and deoxycoformycin starting 20 days after infection with *T.b rhodesiense* contained lower levels of pro-inflammatory cytokine transcripts when studied after 80 days after infection, as compared to non-treated infected groups. Cytokine mRNA levels in infected and cordycepin and deoxycoformycin-treated mice and in uninfected mice were similar. (Figure 6E–H).

### Cordycepin-induced programmed cell death of parasites

**Figure 7:**
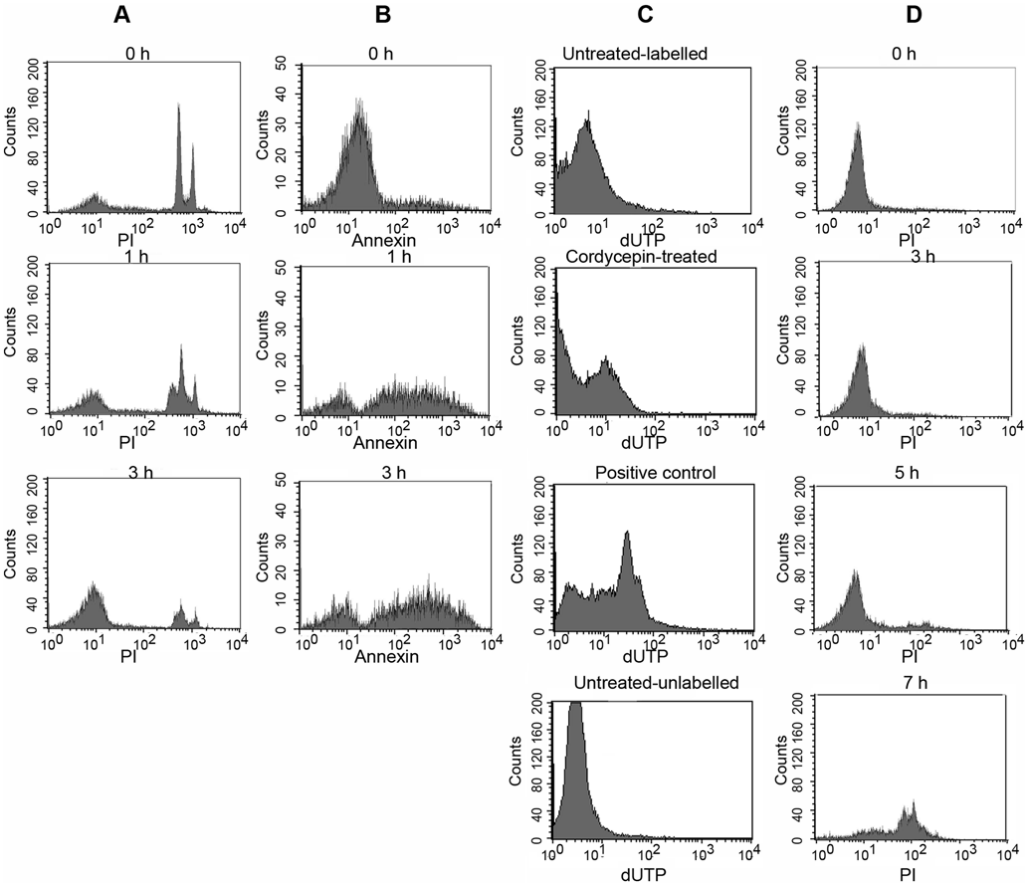
Cordycepin induces programmed cell death of trypanosomes. *T.b.* brucei were treated for the indicated times with 1 µM cordycepin and staining with annexin V, propidium iodide (PI), and Tunel (terminal deoxynucleotidyl transferase-mediated dUTP nick-end labeling) was evaluated by FACS analysis. A *T.b. brucei* were incubated for the indicated time with 1 µM cordycepin, fixed with digitonin and stained with PI. Note presence of subG1 peaks indicative of DNA fragmentation at 1 h after treatment and extensive DNA degradation at 3 h after incubation with cordycepin. B *T.b. brucei* were incubated with 1 µM cordycepin, incubated at 37°C and stained with Annexin V at the indicated time points. C DNA fragmentation in individual fixed *T.b. brucei* before and after incubation with 1 µM cordycepin was visualized by detection of biotinylated nucleotides incorporated onto the free 3′–hydroxyl residues of DNA fragments. Biotinylated nucleotides were then stained with streptavidin-FITC. As a positive control, *T.b. brucei* were fixed, treated with DNAse I and the fragmented DNA was labeled biotinylated nucleotides. Negative controls included cordycepin untreated labeled and unlabeled parasites. D To study necrosis after treatment of *T.b. brucei* with 1 µM cordycepin, nuclei were stained with propidium iodide (5 µg/ml) in the absence of a cell permeant and analyzed by FACS.

The mechanisms accounting for the trypanocidal effect of cordycepin were subsequently explored. Several trypanocidal drugs have been shown to activate a programmed cell death of *T. brucei* [26]. We found that incubation with 1 µM cordycepin induced degradation of DNA by measured by propidium iodide (PI) staining of permeabilized parasites. DNA degradation was detected 1 h after cordycepin treatment and it increased with time (Figure 7A). DNA fragments were also detected by the TUNEL assay in cordycepin-treated parasites (Figure 7C). One of the earliest indications of apoptosis is the translocation of phosphatidylserine from the inner to the outer leaflet of the plasma membrane. Parasites treated with 1 µM cordycepin showed translocation of phosphatidylserine as indicated by the binding of annexin V as early as 1 h after treatment (Figure 7B). On the other hand, no alterations in mitochondrial redox potential of cordycepin-treated parasites were detected (Figure S1; Text S1). DNA degradation occurred in the absence of cell membrane disruption, which could only be detected 7 h after cordycepin treatment (Figure 7D), indicating that cordycepin induced programmed cell death of *T.b. brucei* which was followed by a secondary necrosis.

### Cordycepin-induced drug resistance

**Figure 8:**
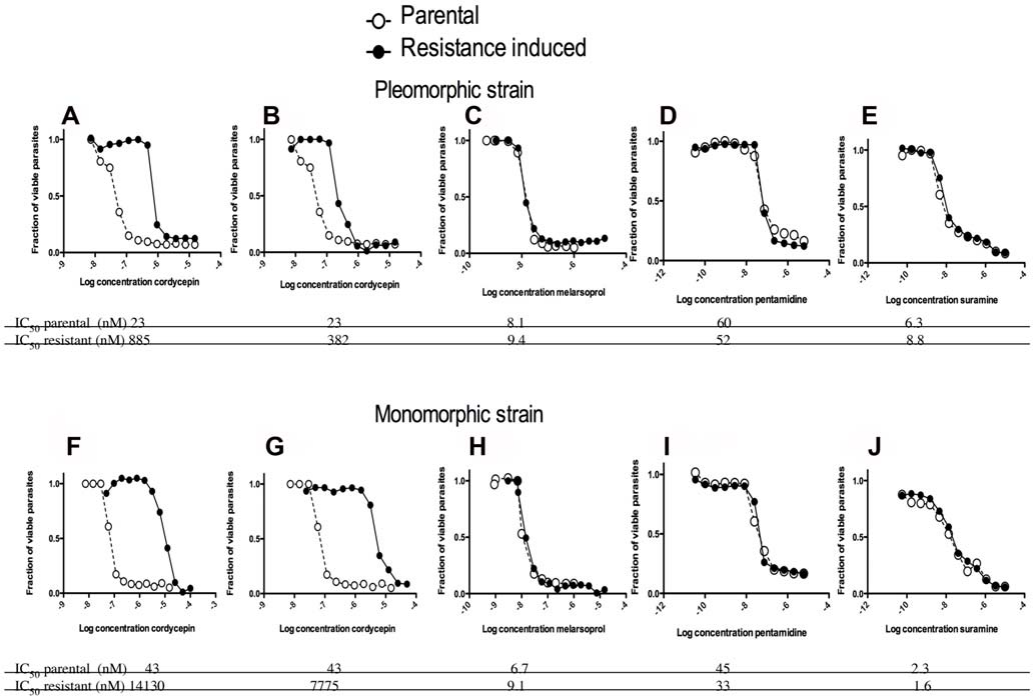
Cordycepin-resistance strains are not cross resistant to suramin, pentamidine and melarsoprol. Cordycepin-resistant pleomorphic AnTat1.1 A–E) and monomorphic Lister 427 F–J) parasites were induced after selection with increasingly higher concentrations of the cordycepin during four months of culture. Parasites in all sub-panels were incubated with the indicated molar concentrations of the trypanocidal compounds for 70 h, when WST-1 reagent added. Two h after incubation, parasite viability was measured. The fraction of parasites in comparison to untreated controls is depicted. A, F Four months after incubation with cordycepin, parasites reached a 40–60 fold decreased sensitivity to cordycepin. B, G Resistance to different concentrations cordycepin after 4 week-long culture of resistant parasites in absence of the drug is shown. Sensitivity of cordycepin-resistant pleomorphic parasites to melarsoprol C, H), pentamidine D, I) or suramin E, J) is depicted.

Whether *T. b. brucei* can develop resistance against cordycepin was then studied. For this purpose *T. b. brucei* AnTat1.1 and Lister 427 were incubated with low concentrations (1 LD_50_ or higher doses) of cordycepin for 72 h at 37°C. Parasites surviving the highest concentrations of cordycepin, were then further cultured for 3 days with similar or higher concentrations of cordycepin. This procedure was repeated for four months, after which a 40–60 fold increased resistance to cordycepin, compared to the parental strain was achieved (Figure 8A, F).

Resistance to the nucleoside analogue was retained after culture of the resistant parasites for three weeks in absence of cordycepin, suggesting that resistance was due to genetic alterations rather than to a metabolic adaptation of the parasite (Figure 8 B, G). Cordycepin-resistant parasites were then incubated with melarsoprol, pentamidine and suramine to determine cross-resistance. No cross-resistance with these trypanocidal drugs was observed, indicating that these trypanocidal compounds have non-overlapping molecular targets (Figure 8C–E, H–J).

**Figure 9:**
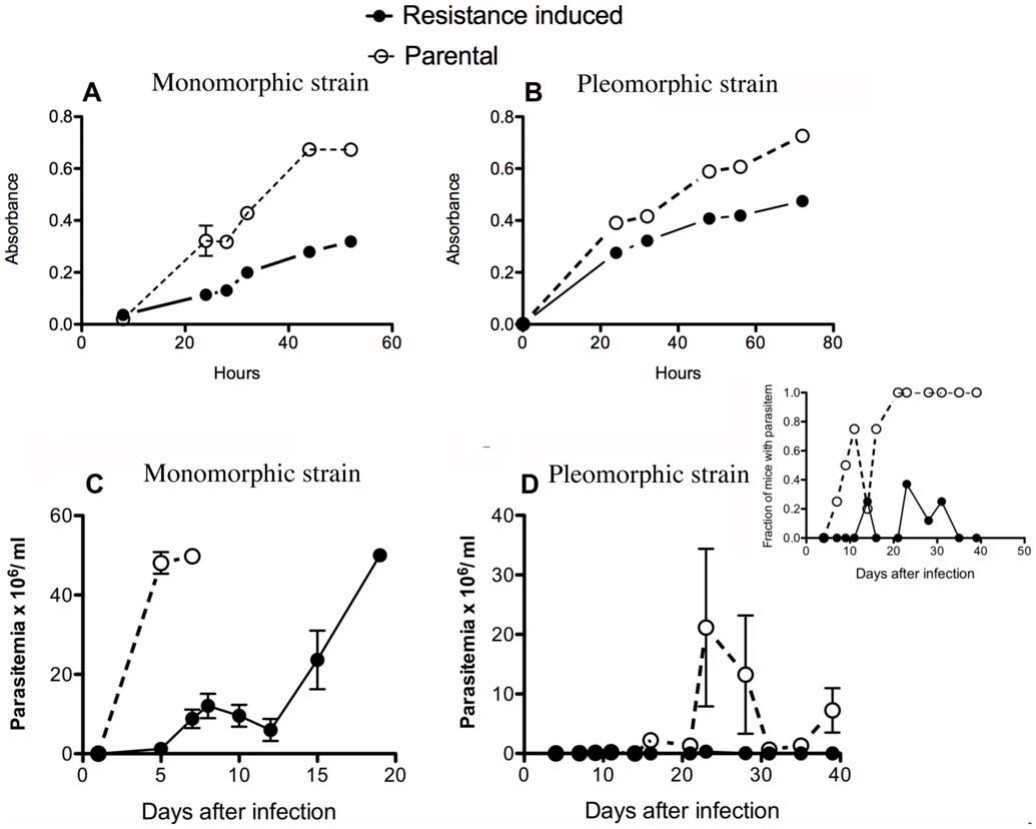
Cordycepin-resistant parasites showed diminished growth in vitro and diminished virulence in vivo. A, B The growth of resistant-induced or parental parasites were measured by WST-1 oxidation at different time points after incubation of similar numbers of resistance-induced and the parental monomorphic A) or pleomorphic B) parasites. The doubling times for the monomorphic parental population was 5.6 h and 14.6 h for the resistant strain. The doubling time for the pleomorphic strain was 27 h and 45 h for the cordycepin-resistant parasites. C, D Parasitemia of mice inoculated i.p. with 2×10^4^ resistant-induced or parental *T.b. brucei* monomorphic C) or pleomorphic D) strains. The insert to figure D shows that despite lower level of parasitemia in comparison to parental strains, parasitemia can be registered in a fraction of mice infected with the pleomorphic cordycepin-resistant strain.

Cordycepin-resistant strains displayed lower growth *in vitro* compared to their respective parental strains (Figure 9A, B). *In vivo*, mice inoculated with cordycepin-resistant parasites showed delayed and lower parasitemia levels compared to controls (Figure 9C, D). Parasitemia in mice inoculated with cordycepin-resistant AnTat1.1 became undetectable. Loss of weight was registered in mice infected with the parental strains but this effect was reduced in mice infected with cordycepin resistant strains (data not shown).

### Sequence analysis of candidate target genes

In order to determine possible cordycepin targets we amplified, cloned and sequenced 11 candidate genes involved in uptake and salvage of adenosine analogues in resistance-induced and parental parasites. The TbAT1, TbNT2, TbNT4, TbNT6, TbNT12 nucleoside transporter genes, TbHT1 hexose transporter, TbAK adenosine kinase, TbHGPRT hypoxanthine-guanine phosphoribosyltransferase from parental and resistant strains were screened for mutations that could cause an affected or deleterious protein function.

The HT1 was selected since this hexose transporter can be inhibited by tubericidin [24]. TbNT2 is predominantly expressed when compared to other genes in P1 family. The expression of TbNT3, TbNT4, TbNT5, TbNT7, is not seen in a parental *T. b. brucei* strtain. It has been reported that loss of primary transporter P2 leads to over expression of TbNT4 and TbNT6 [27]. TbNT2, 4, 6 and 12 were therefore selected for sequencing.

**Figure 10:**
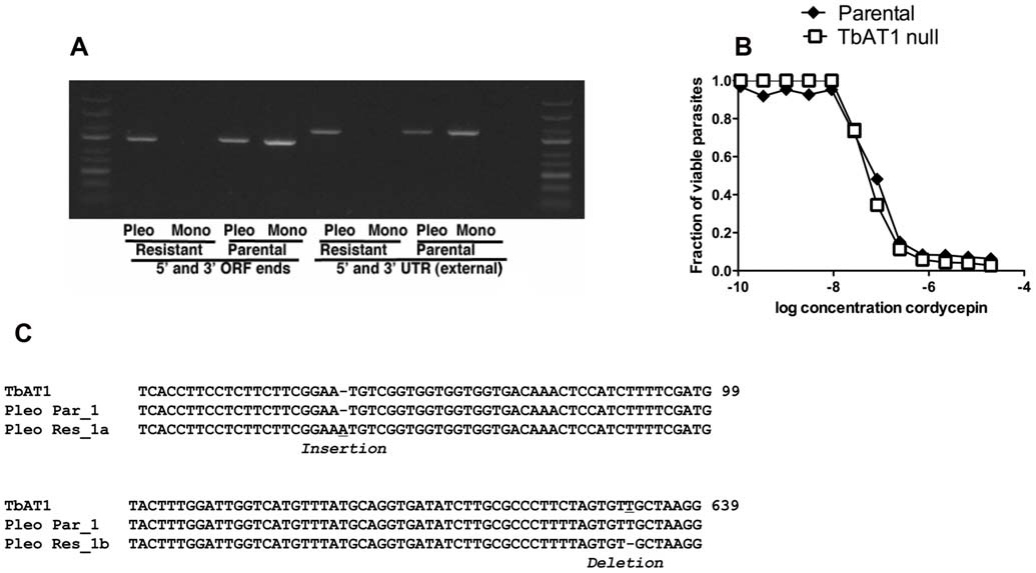
Sequencing of candidate genes. A) The PCR DNA amplification from *T.b. brucei* pleomorphic AnTat1.1 or monomorphic Lister 427 parasites is shown. Primers recognizing the 5′ and 3′ UTR and the 5′ and 3′ ends of the ORF of the TbAT1 gene were used. B) A single base insertion at position 61 and a single base deletion at position 631, causing the synthesis of a non-functional truncated gene product in clones of the resistant pleomorphic strain but not in the parental or in the genome annotated strain (Lister 427). C) TbAT1^−/−^ and parental parasites were incubated with different doses of cordycepin for 70 h, when WST-1 reagent added. Two h after incubation, parasite viability was measured. The fraction of parasites in comparison to untreated controls is depicted.

While several polymorphisms were found, no deleterious mutations were found in the genes sequenced except for TbAT1. TbAT1 is a nucleoside adenosine transporter present in the genome of *T. brucei* as a 1392 bp single copy gene where both alleles have been found to be almost identical. We found two mutations in the cordycepin-resistant AnTat1.1 strain TbAT1 gene that were not present in the respective parental DNA. There was a single base insertion at position 61 and a single base deletion at position 631, that could cause synthesis of a non-functional truncated gene product were found (Figure 10B). Five out of 12 sequenced colonies presented the one base insertion while the other seven carried the deletion, both groups with a read coverage of 15×, which confirmed the mutations. We assume that these two sequences detected correspond to the two alleles of TbAT1 carrying different mutations but rendering the same non-functional transporter protein.

For the cordycepin-resistant monomorphic Lister 427 strain, PCR using primers designed at the 5′ and 3′ ends of the gene and at the 5′ and 3′ UTRs were unable to amplify any product (Figure 10A), while the same set of primers amplified the TbAT1 gene from the parental DNA. We assume that in this case the mutations on this gene probably generated the deletion of a partial or the complete gene sequence, as amplification with internal primers although producing a PCR fragment, was not of the expected size (data not shown).

Since cordycepin resistant monomorphic (Lister 427) and pleomorphic (AnTat1.1) parasites displayed mutations in the TbAT1 gene, we tested whether this transporter affected the susceptibility of parasites to cordycepin by using TbAT1 knockout parasites. We found that resistance of cordycepin by knockout parasites was only slightly affected by the mutation, suggesting that the absence of the P2 transporter is not sufficient to explain the observed increased resistance of parasites to cordycepin (Figure 10C).

## Discussion

We here report a preclinical evaluation of the chemotherapeutic efficiency of cordycepin and deoxycoformycin for late stage infection with *T. brucei*. In brief, cordycepin was selected from a direct parasite viability screening of a compound library of nucleoside analogues. The minimal effective concentrations of cordycepin and deoxycoformycin for late stage treatment *in vivo* were determined. Of importance, the dose of deoxycoformycin used is similar or lower to that employed in leukemia patients, and cordycepin and deoxyformycin have been tested in clinical trials in humans (R McCaffrey, Harvard Medical School, Boston, personal communication) and in preclinical studies in dogs at higher concentrations to the ones here presented [28]. The compounds could be given i.p., s.c. and orally, and our data suggests that a slow release of the compounds *in vivo* will improve efficacy of treatment by reducing the doses, and that enteric coating might reduce the problem of acid lability of deoxycoformycin. The nucleoside analogue treatment was also effective for treatment of late stage infections with both *T.b. rhodesiense* and *T.b. gambiense*, different to some drugs in clinical use such as DFMO (eflornithine), which is only effective against *T.b. gambiense*. Cordycepin and deoxycoformycin treatment diminished the inflammatory cytokine accumulation in the brains of infected mice. In agreement, the number of CD45+ inflammatory cells in the brain parenchyma of *T. brucei*-infected mice was also reduced after treatment cordycepin and deoxycoformycin [15]. We found standard parameters associated to programmed cell death in *T. brucei* to be similar to those described in pluricellular organisms. Programmed cell death was followed by secondary necrosis. Interestingly, programmed cell death processes have previously been shown to be activated during the life cycle of trypanosomatids, regulating parasite number in certain host tissues, in the maintenance of clonality, as a mechanism of immunomodulation and of parasite differentiation [29]. Cordycepin thus stimulates a natural process of cell death, suggesting the presence of parasite cordycepin-sensitive targets regulating such processes.

The development of this new drug combination with reduced toxicity, high activity and oral availability gives rise to renewed hope of new and safer treatments of HAT. However, the utility and longevity of these new treatments will, to a large degree, be determined by the development of parasite resistance to the drug. We found that although *T.b. brucei* developed resistance to cordycepin upon prolonged culture with low doses of the compound, such resistant parasites showed diminished virulence and reduced growth *in vivo*. Moreover, resistant parasites showed no cross resistance to melarsoprol, suramin and pentamidine in clinical use for early or late stage treatment of HAT, suggesting that cordycepin targets are different to those of the other drugs. This is of importance since a strategy to delay or prevent the development of resistance is to combine drugs that act on different targets and therefore are unlikely to show cross-resistance. Additionally, synergistic effects may arise from a combination of two drugs that act differently. In such a situation, the concentrations of the combination partners can be reduced compared with those used in a mono-therapy, which leads to better safety.

Since resistance to cordycepin was slowly increased during the four months-long culture of the parasites with the drug (data not shown), it is likely that a number of mutations had accumulated during the process. After cloning and sequencing 11 candidate genes from cordycepin-induced resistant and parental strains, we found that parasites showed altered functional mutations only in the P2 transporter encoded by the TbAT1 gene. Although mutations found in the other examined genes were present, they did not show any predicted deleterious or altered effect for the respective protein function. Cordycepin uptake has been reported to be mediated by both the P1 and P2 transporters [27]. We found however that a TbAT1 (P2) null parasite showed only slight reduction in resistance to cordycepin, suggesting that other undefined mutated genes also participate in increased sensitivity to cordycepin of parental parasites.

Since cordycepin-resistant parasites show a genetic defect in TbAT1, it was surprising that no cross resistance to pentamidine, suramin and melarsoprol was not observed: the P2 transporter has been shown to be responsible for uptake of different trypanocides such as melaminyl arsenicals, pentamidine, diminazenes, cordycepin and tubercidin [30],[31],[32],[33]. Homozygously disrupted TbAT1 null *T. brucei* showed two- and three-fold increases resistance to melarsoprol and melarsen [17].

Most drugs for treatment of HAT were developed in the first half of the twentieth century, and some of them would not pass current high safety standards. A safe drug that is effective in the treatment of the encephalitic stage of HAT would significantly change the control and management of sleeping sickness. The data here presented strongly suggest that the combination of cordycepin and deoxycoformycin can be used in clinical tests for treatment of African trypanosomiasis. Besides the results from the preclinical experiments described in our manuscript, cordycepin fulfills a number of requirements for to be considered a candidate drug: It is available for other purposes and cheap, it can be given orally, clinical trials of the doublet have been performed and toxicity in man at the same concentrations used in animals seems not to pose a problem.

## Supporting Information

Alternative Language Abstract S1 Spanish translation of the abstract by MER.
(0.04 MB DOC)

Figure S1 The depolarization of mitochondrial membrane in *T.b. brucei* incubated with 1 µM cordycepin was measured by incubating *T. brucei* with TMRE.
(0.59 MB PPT)

Table S1 Sequencing primers list
(0.06 MB DOC)

Text S1 Supplementary methods.
(0.03 MB DOC)
