# Supplementary material for: Preclinical Assessment of the Treatment of Second-Stage African Trypanosomiasis with Cordycepin and Deoxycoformycin
Source: PLoS Negl Trop Dis. 2009 Aug 4;3(8):e495. doi: 10.1371/journal.pntd.0000495 (PMC2713411; doi:10.1371/journal.pntd.0000495)
Supplement: Alternative Language Abstract S1 — Spanish translation of the abstract by MER. (0.04 MB DOC) [file pntd.0000495.s001.doc]

*Translation of Abstract into Spanish by M. Rottenberg*

Resumen

*Antecedentes*

Existe una imperiosa necesidad de sustituir las drogas en uso para el tratamiento de la fase neurológica o segunda fase de la tripanosomiasis africana humana (TAH), ya que estas drogas son altamente tóxicas. En este trabajo hemos evaluado el tratamiento de esta fase de la infección por *Trypanosoma brucei* con cordicypina y deoxycoformycina, un inhibidor de la adenosina deaminasa.

*Metodología y hallazgos principales*

La cordycepina fue seleccionada por su capacidad tripanocida como el mas eficiente de una biblioteca de 2200 nucleósidos. El número de dosis y las concentracones minimas de los compuestos capaces de tratar la segunda fase de a infección murina por *T. brucei* fue determinada. Tanto el tratamiento oral como el subcutáneo o el intraparentoneal fueron efectivos. La administración de cordycepina y deoxycoformycina curó ratones durante la segunda fase de la infección por las subespecies patogénicas humanas *T. b. rhodesiense* y *T. b. gambiense*. El tratamiento con el doblete en la segunda fase de infección disminuyó notablemente la inflamación cerebral.

La cordycepina indujo la muerte celular programada, seguida por la necrosis secundaria de los parásitos. *T. b. brucei* desarrollaron gradualmente resistencia a la cordycepina al ser incubados con bajas dosis de este compuesto. Sin embargo, los parásitos resistentes sufrieron una significativa pérdida de virulencia y no mostraron resistencia cruzada con drogas tripanocidas en uso: melarsoprol, suramina or pentamidina. A pesar que los parásitos resistentes a cordycepina sufrieron mutaciones amórficas en el gen que codifica para el receptor de adenosina P2, parásitos genómicamente delecionados de este gen no mostraron un aumento de la resistencia a la cordycepina, indicando que la ausencia de este transportador no es suficiente para inducir un amento en la resistencia a la cordycepina.

*Conclusiones/ relevancia.*

Nuestros datos apoyan fuertemente el desarrollo de estudios clínicos utilizando cordycepina y deoxycoformycina como tratamiento alternativo de la fase neurólogica de la tripanosomiasis humana africana o en formas resistentes a melarsoprol.
