# Supplementary figures and images for: Preclinical Assessment of the Treatment of Second-Stage African Trypanosomiasis with Cordycepin and Deoxycoformycin
Source: PLoS Negl Trop Dis. 2009 Aug 4;3(8):e495. doi: 10.1371/journal.pntd.0000495 (PMC2713411; doi:10.1371/journal.pntd.0000495)

## Slide 1
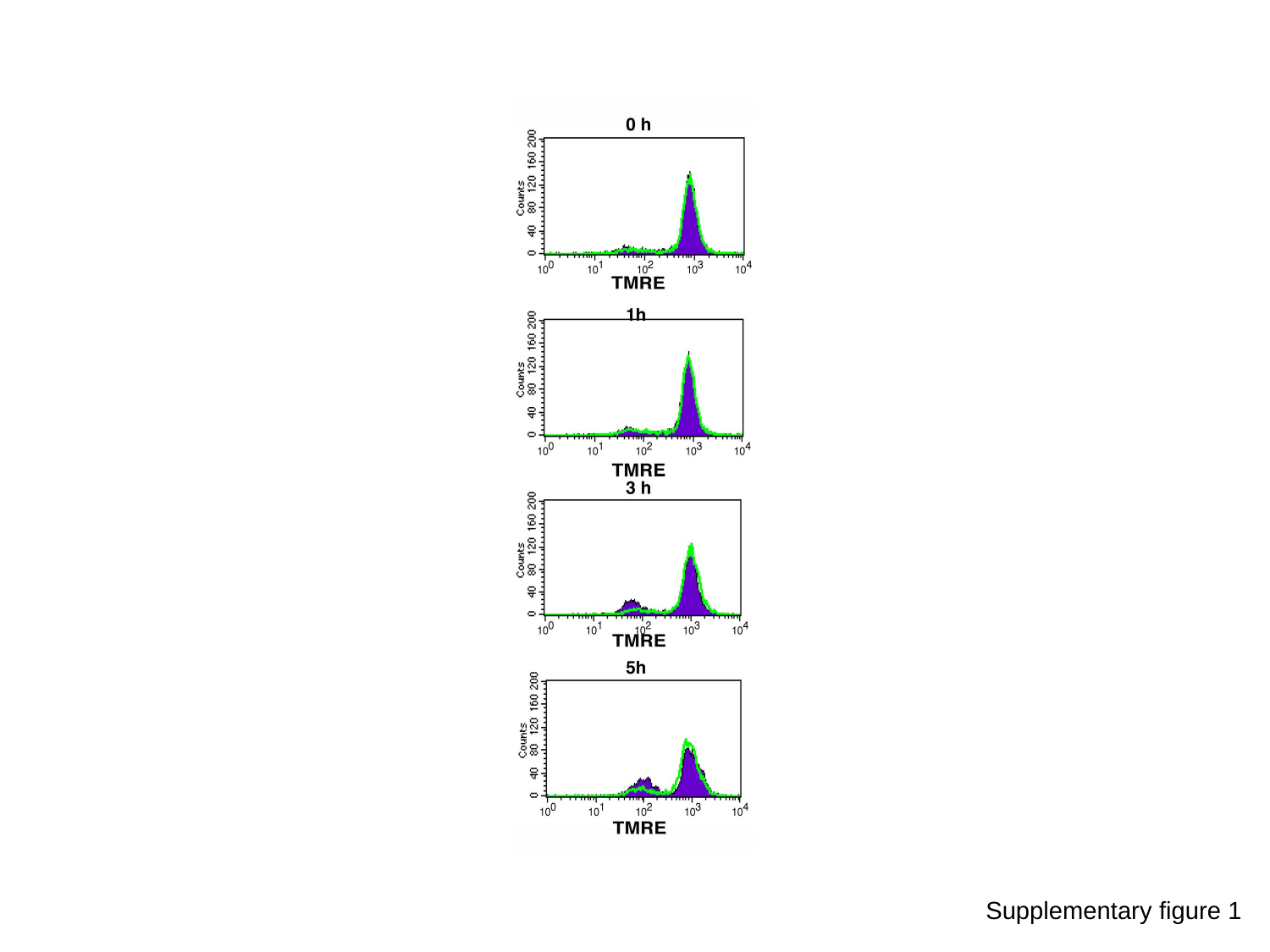

Supplementary figure 1

Supplement: Figure S1 — The depolarization of mitochondrial membrane in T.b. brucei incubated with 1 µM cordycepin was measured by incubating T. brucei with TMRE. (0.59 MB PPT) [file pntd.0000495.s002.ppt]
