# Supplementary material for: Preclinical Assessment of the Treatment of Second-Stage African Trypanosomiasis with Cordycepin and Deoxycoformycin
Source: PLoS Negl Trop Dis. 2009 Aug 4;3(8):e495. doi: 10.1371/journal.pntd.0000495 (PMC2713411; doi:10.1371/journal.pntd.0000495)
Supplement: Table S1 — Sequencing primers list (0.06 MB DOC) [file pntd.0000495.s003.doc]

Table S1

Sequencing primers list

| Gene | Accession number | Forward | Reverse |
| --- | --- | --- | --- |
| **Nucleoside transporter**  **(TbNT2)** | XM_946653 | ATGGCAATGCTTGGT TTCGAGTCG | CTACTTTTTTTCCTTCGG AAGTCCCTCC |
| **Nucleoside transporter**  **(TbNT4)** | XM_946657 | ATGGCAATGCTTGGTTTC GAGTCG | CTACTTTTTTTCCTTCGG AAGTCCCTCC |
| **Nucleoside transporter (TbNT6)** | XM_946663 | ATGGCAATGCTTGGT TTCGAGTCG | TTATTTAGGAAGTCCCTC CTTAACAGCC |
| **Nucleoside transporter (TbNT12)** | XM_838562 | ATGGCAATGCTTGGTTTC GAG TCG | CTACTCTGTTTTAGGAAG AGCCTCC |
| **Nucleoside transporter (TbAT1)** | Tb927.5.286b | ATGCTCGGGTTTGACTCA GC | CTACTTGGGAAGCCCCTC ATTG |
| **Adenosine kinase** | XM_840258  XM_840264 | ATGTCATCC GCT CCT CTG AGGGTATACG | TCA AGGAGAGAAACT GGGCTT CTC GGG G |
| **Adenine phosphoribosyltransferase** | XM_840709 | ATG TCACTT GTGGAGGTT TTGCC | TTAATCAATTTTTT TGCCTC GGCGCAG |
| **Adenine phosphoribosyltransferase** | XM_840710 | ATGTCACAGTATGATGCG ATTCTGAC | TCACAGTCGTGACCTGGT AATGTATG |
| **Hypoxanthine guanine phosphoribosyltransferase** | XM_817304;  XM_817305; | ATGGAACCAGCTTGC AAATACGACTTC | TTACAGTTTTGCCTT CAC AGCGG |
| **Hypoxanthine guanine phosphoribosyltransferase** | XM_817312 | ATGCACTCGGGCCATCCT CTCAAACC | TTACAATTTGCTCGGGTA CCGTTTCGC |
| **Hexose transporter** | XM_81798; XM_817986 | ATGACTGAGCGTCGTGAT AACGTTTCC | TTAGTTCCGCGGAGATGC TTC |
| **UTR of TbAT1** | **Tb927.5.286b** | TTG TTT CCG TTA CAA ATC CCC GTT CC | TGG CGA ATC GGT GTA CGT TAA CG |
