# Supplementary material for: Preclinical Assessment of the Treatment of Second-Stage African Trypanosomiasis with Cordycepin and Deoxycoformycin
Source: PLoS Negl Trop Dis. 2009 Aug 4;3(8):e495. doi: 10.1371/journal.pntd.0000495 (PMC2713411; doi:10.1371/journal.pntd.0000495)
Supplement: Text S1 — Supplementary methods. (0.03 MB DOC) [file pntd.0000495.s004.doc]

**SUPPLEMENTARY METHODS**

*Mitochondrial membrane potential*

The lipophilic TMRE dye, enters the mitochondrion where it accumulates in an inner-membrane potential-dependent manner. When the mitochondrial collapses in apoptotic cells, the dye no longer accumulates inside the mitochondria and becomes evenly distributed throughout the cytosol, and overall cellular fluorescence levels drop. Mitochondrial membrane potential was measured after incubation of cordycepin-treated and untreated *T.b. brucei* with 25 nM TMRE for 30 min at 37°C followed by FACS analysis detected with FL2 emmission as described [18].
